# Characterization and PCR Application of Family B DNA Polymerases from *Thermococcus stetteri*

**DOI:** 10.3390/life14121544

**Published:** 2024-11-25

**Authors:** Aleksandra A. Kuznetsova, Marina A. Soloveva, Elena S. Mikushina, Anastasia A. Gavrilova, Artemiy S. Bakman, Nikita A. Kuznetsov

**Affiliations:** 1 Institute of Chemical Biology and Fundamental Medicine, Siberian Branch of Russian Academy of Sciences, Novosibirsk 630090, Russia; m.soloveva2@g.nsu.ru (M.A.S.); elenamikushina01@mail.ru (E.S.M.); anaroslakova@gmail.com (A.A.G.); art-bakman@yandex.ru (A.S.B.); 2 Department of Natural Sciences, Novosibirsk State University, Novosibirsk 630090, Russia

**Keywords:** PCR, *Thermococcus stetteri*, fusion DNA polymerase, mutagenesis, enzyme activity, family B, native enzyme

## Abstract

DNA polymerases from the hyperthermophilic Archaea have attracted considerable attention as PCR enzymes due to their high thermal stability and proofreading 3′ → 5′ exonuclease activity. This study is the first to report data concerning the purification and biochemical characteristics of the Tst DNA polymerase from *Thermococcus stetteri*. Both the wild type Tst(wt) DNA polymerase and its chimeric form containing the P36H substitution—which reduces the enzyme’s affinity for the U-containing template and dUTP—and the DNA-binding domain Sso7d from *S. solfataricus* were obtained and analyzed. It was shown that Tst(wt) could effectively amplify up to 6-kb DNA fragments, whereas TstP36H–Sso7d could amplify DNA fragments up to 15 kb. It was found that TstP36H–Sso7d has superior PCR efficiency compared to the commonly used DNA polymerase PfuV93Q–Sso7d. For the amplification of a 2-kb DNA fragment, TstP36H–Sso7d required less than 10 s of extension time, whereas for PfuV93Q–Sso7d, the extension time was no less than 30 s. Steady-state kinetic assays revealed that the dNTP-binding affinity *K*^dNTP^_m_ was the same for TstP36H–Sso7d and PfuV93Q–Sso7d, whereas the maximum rate of dNTP incorporation, *k*_cat_, was two orders of magnitude higher for TstP36H–Sso7d. Moreover, the incorporation of incorrect dNTP was not observed for TstP36H–Sso7d up to 56 °C, whereas for PfuV93Q–Sso7d, the extension of primer with incorrect dNTP was observed at 37 °C, supporting higher fidelity of TstP36H–Sso7d. The obtained data suggest that TstP36H–Sso7d may be a good candidate for high-fidelity DNA amplification.

## 1. Introduction

DNA polymerase (EC number 2.7.7.7) plays a major role in molecular biological applications like DNA amplification and sequencing. For rapid amplification of selected DNA segments, PCR is used [1]. The use of high-fidelity DNA polymerases is important for minimizing nucleotide misincorporation errors in amplified products; moreover, the high temperatures needed for PCR require a thermostable enzyme. Numerous thermostable DNA polymerases have been described and commercialized for the PCR technique [2]. All of these belong mainly to A-type and B-type polymerases, namely bacterial Pol I-type enzymes and archaeal Pol II-type enzymes.

Each DNA polymerase is characterized by its own set of unique properties, including the extension rate, fidelity, thermostability, processivity, specificity, resistance to inhibitors and contaminants, modified nucleotide selection, ability to bypass damage, etc. The distinctive properties can be used to create unique enzymes [3,4,5,6,7]. Despite the number of known and available enzymes, the increasing number of applications in molecular biology has led to a growing demand for novel enzymes, and from this point of view, the search for novel DNA polymerases has been an important focus over the past few decades. A-type polymerases from the genus *Thermus* are frequently used in molecular biology, including the commonly used Taq DNA polymerase from *T. aquaticus*. DNA polymerases with similar properties to Taq have been described from other *Thermus* species [8], including Tfi from *T. filiformis* [9], Tfl from *T. flavus* [10], Tca from *T. caldophilus* [11], Tth from *T. thermophiles* [12], and TsK1 from *T. scotoductus* [13]. Other A-type polymerases have been isolated from *Thermotoga* spp., including Tma polymerase from *T. maritima* [14] and Tne from *T. neapolitana* [15], as well as DNA polymerase Aae from *Aquifex aeolicus* [16].

Family A DNA polymerases have general drawbacks concerning a relatively high error frequency in the final amplified product. Thus, family B DNA polymerases from the hyperthermophilic Archaea have attracted considerable attention as PCR enzymes due to their high thermal stability and proofreading 3′ → 5′ exonuclease activity. A large number of DNA polymerases belonging to the B family have been characterized, especially from the *Thermococcales* order, including Pfu from *Pyrococcus furiosus* [17], Pab from *Pyrococcus abyssi* [18], Pwo from *Pyrococcus woesei* [19], KOD1 from *Thermococcus kodakaraensis* [20], Tli from *Thermococcus litoralis* [21], Tfu from *Thermococcus fumicolans* [22], Tce from *Thermococcus celer* [23], Twa from *Thermococcus waiotapuensis* [24], Tgo from *Thermococcus gorgonarius*, Tpa *Thermococcus pacificus* [25], and others [26,27,28,29].

Slight differences in amino acid sequences between DNA polymerases can result in dramatic effects on their biochemical characteristics, demonstrating that it is possible to find novel polymerases with improved functionality. Here, we describe the expression, purification, and characterization of a recombinant DNA polymerase from *Thermococcus stetteri* [30] (Tst DNA polymerase) originating from marine hydrothermal vents in the Kuril Islands region. This enzyme has been shown to have higher fidelity and processivity when compared to PfuV93Q–Sso7d, making it attractive for molecular biology applications.

## 2. Materials and Methods

### 2.1. Origins of the Archaeal Strain, Laboratory E. coli Strains, and Enzymes

The *Thermococcus stetteri* strain was obtained from the All-Russian Collection of Microorganisms (VKM, Pushchino, Russia). *E. coli* strain DH5a (Thermo Scientific, Waltham, MA, USA) was used for plasmid propagation while *E. coli* strain Rosetta2 (DE3)pLysS (Novagen, Merck KGaA, Darmstadt, Germany) was used for gene expression. T4 DNA ligase was obtained from SibEnzyme (Novosibirsk, Russia). Restriction endonucleases FoundI and CCiNI are isoschizomers of NdeI and NotI, respectively, and were obtained from SibEnzyme (Novosibirsk, Russia). PfuV93Q–Sso7d DNA polymerase, sourced from our own laboratory collection, was used for PCR procedures and served as a comparison enzyme in the investigation of the biochemical properties of the Tst DNA polymerase.

### 2.2. Molecular Cloning

The gene-encoding full-length DNA polymerase I Tst was amplified from *T. stetteri* strain genomic DNA (GenBank ID: MBP1912549.1). DNA manipulation and *T. stetteri* genomic DNA isolation were performed using standard procedures. For amplification of the Tst DNA polymerase gene, the following primers were used: a forward primer with the FoundI restriction site and a reverse primer with the CciNI restriction site (Table 1). PCR reactions were performed in a total volume of 50 µL containing the following PCR mixture: 1× PfuSE buffer (SibEnzyme, Novosibirsk, Russia), 400 μM of dNTPs, 200 nM of each primer, 5% dimethyl sulfoxide, and 2 units of PfuV93Q–Sso7d. PCR reactions were performed with an initial denaturation at 94 °C for 4 min, 30 cycles of denaturation (94 °C, 30 s), annealing (60 °C, 30 s), and extension (72 °C, 2 min), followed by a final extension at 72 °C for 10 min. The amplified 2.7-kb PCR product was then digested with FoundI (SibEnzyme, Novosibirsk, Russia) and CciNI (SibEnzyme, Novosibirsk, Russia), purified from 0.8% low-melting agarose gel using the Cleanup S-Cap kit (Evrogen Joint Stock Company, Moscow, Russia), and then inserted into the pET28c (Novagen) expression vector according to the manufacturer’s protocol with T4 DNA ligase (SibEnzyme, Novosibirsk, Russia). The ligation mixture was then used to transform *E. coli* strain DH5a. Clones with the correct construct were selected through restriction enzyme analysis and sequencing.

Point mutation P36H was introduced into the Tst DNA polymerase gene by site-directed mutagenesis using primers with specific nucleotide substitutions at the P36 residue (Table 1). Also, a chimeric form of Tst P36H DNA polymerase with DNA-binding protein Sso7d from *Sulfolobus solfataricus* was obtained by site-directed mutagenesis, which involved the insertion of 207nt nucleotide sequence coding for Sso7d before the stop codon (Table 1). All variants of the modified Tst DNA polymerase were synthesized via PCR amplification, and subsequently digested with the restriction enzyme MalI (SibEnzyme, Novosibirsk, Russia) to digest non-mutated parental methylated plasmid DNA. The mutated plasmids were transformed into *E. coli* DH5a for nick repair. The introduced mutations were confirmed by sequencing.

### 2.3. Expression and Purification of Recombinant Tst DNA Polymerases

*E. coli* strain Rosetta2 (DE3)pLysS (Novagen, Merck KGaA, Darmstadt, Germany) was transformed with pET28c–Tst(wt) or pET28c–TstP36H–Sso7d and was grown at 37 °C in 15 mL of LB Broth containing 50 mg/mL kanamycin O.N. as the starter. The starter was added to 1 L of LB Broth containing 50 mg/mL kanamycin, and cells were grown with shaking at 37 °C to an OD_600_ of 0.6–0.8. Overexpression of the DNA polymerase genes was induced by the addition of isopropyl β-D-1-thiogalactopyranoside (IPTG) to a final concentration of 0.2 mM, followed by an additional 20 h of growth at 20 °C. The cells were harvested via centrifugation at 4000× *g* for 20 min at 4 °C and resuspended in 20 mM HEPES–KOH buffer (pH 7.8), 40 mM NaCl. The cells were disrupted by the French press, and cellular debris was removed via centrifugation at 40,000× *g* for 40 min at 4 °C. To the resulting supernatant, a solution of NaCl and imidazole was added to concentrations of 500 and 20 mM, respectively. The resulting solution was mixed with 1 mL of the Ni Sepharose^TM^ High-Performance resin (Amersham Biosciences, Uppsala, Sweden) and stirred for 1 h at 4 °C. The enzyme was eluted with 5 mL of a buffer consisting of 20 mM HEPES–NaOH pH 7.8, 500 mM NaCl, and 600 mM imidazole. The obtained enzyme-containing fraction was diluted to a final NaCl concentration of 40 mM and applied to a HiTrap Heparin™ column (Amersham Biosciences, Uppsala, Sweden) at a flow rate of 0.4 mL/min. The chromatography was performed in a buffer containing 20 mM HEPES–NaOH and a linear 40 → 1000 mM gradient of NaCl; the optical density of the solution was recorded at 280 nm. For TstP36H–Sso7d, the third stage of purification was performed, namely the reversed-phase chromatography. To the enzyme-containing fractions, a solution of 5 M NaCl was added to achieve a final concentration of 3 M, and a resulting solution was applied to a HiTrap Phenyl HP^TM^ column (Sepax Technologies, Inc., Newark, DE, USA) at a flow rate of 0.4 mL/min. The chromatography was performed in a buffer containing 20 mM HEPES–NaOH, with a linear 3 M → 0 gradient of NaCl. 

The purity of the Tst DNA polymerases was determined via gel electrophoresis. Fractions containing the DNA polymerases were collected and glycerol solution was added to make up 50% of the volume. The enzyme was stored at −20 °C. The protein concentration was determined using the Bradford assay, and protein purity was examined using sodium dodecyl sulfate–polyacrylamide gel electrophoresis (SDS–PAGE).

### 2.4. DNA Polymerase Unit Assay

The DNA polymerase activity of the purified enzyme was determined according to [31]. The basic reaction mixture (50 µL) contained 50 mM Tris–HCl (pH 7.4), 5 mM MgCl_2_, 50 mM KCl, 100 µM each of dATP, dTTP, dCTP, dGTP, a 60 nM DNA substrate, and an enzyme solution (0.1–10 nM). The DNA substrate was an annealed 90nt template/33nt primer (Table 2). The template/primer complex was extended with Tst DNA polymerases at 72 °C; subsequent quenching was performed with 0.5 M EDTA to achieve a final concentration of 50 mM, at a set range of time points. A 20× EvaGreen^®^ solution («Biotium», Fremont, CA, USA) was added to each probe to achieve a final 1× concentration. EvaGreen^®^ bound to double-stranded DNA, leading to a significant increase in its fluorescence intensity. The mixtures were incubated at 25 °C for 2 min, and the fluorescence intensity was measured (λ_em_ = 526 nm) using a Cary Eclipse spectrofluorometer (Agilent Technologies, Santa Clara, CA, USA). The reactions experienced an initial linear phase, and the reaction rate depended on the amount of enzyme added. The initial velocities (in pmol/min) of deoxynucleotide incorporation were calculated [31]. One unit of polymerase activity was defined as the amount required to catalyze the incorporation of 10 nmol of dNTPs at 72 °C in 30 min. 

### 2.5. Thermostability Assay

The thermal stabilities of the polymerases were analyzed via heating samples using QuantStudio 5 (Thermo Fisher Scientific, Waltham, MA, USA) with ProteOrange Protein Gel Stain (Lumiprobe Corporation, Hunt Valley, MD, USA); any increase in fluorescence was measured. Thermal melts were carried out in 20 μL of 40 mM HEPES–KOH, 400 mM NaCl (pH 7.0), containing 5× ProteOrange (5000× stock in dimethylsulfoxide), 2 M of guanidine chloride, and 8 μM of Pfu mutant forms. Excitation and emission values were 470 nm and 555 nm, respectively. The temperature increased from 25 to 60 °C at a rate of 0.4 °C per second and from 60 to 99.9 °C at a rate of 0.015 °C per second. Data analysis was carried out with QuantStudio^TM^ Design & Analysis software v1.5.2.

### 2.6. Optimization of PCR Amplification

Oligonucleotide primers that anneal to λ phage DNA were used in the PCR assays (Table 2). PCR buffer optimization experiments were performed using 2 U of Tst(wt) DNA polymerase in a 50 µL reaction containing 0.2 pmol of each primer (λ–Anchor–F and λ–2 kb–R), 1.25 mM of dNTPs, and 50 ng of λ phage DNA as a template. PCR reactions were performed with an initial denaturation at 94 °C for 4 min, 30 cycles of denaturation (94 °C, 30 s), annealing (60 °C, 30 s), and extension (72 °C, 2 min).

To determine the optimal pH, two buffer systems with different buffering capacities (Tris–HCl [pH 7.6–9.0], and glycine–NaOH [pH 9.2–10.6] at 25 °C) were tested. The influences of KCl, MgCl_2_, and (NH_4_)_2_SO_4_ on polymerase activity were determined by adding various concentrations of the respective compound to the basic reaction mixture. To determine the optimal temperature, the temperature of the extension stage was varied (60–85 °C). The optimal reaction buffer (reaction buffer1) achieved 20 mM of Tris–HCl (pH 8.4), 20 mM of KCl, 3 mM of MgCl_2_, 12.5 mM of (NH_4_)_2_SO_4_), and 1.25 mM of dNTPs. All measurements were carried out in triplicate.

To investigate the thermostability properties of Tst(wt), we subjected the purified enzyme, without any additional stabilizers, to 95 °C for up to 24 h. Aliquots of the enzyme were removed at 1, 2, 3, 4, 5, 6, 7, 8, 10, and 24 h, and placed on ice for 2 min. The residual activities of these samples were then determined in the optimal reaction buffer1 using the DNA 44-mer template (Template_44nt), annealing to the 24-mer FAM 5′ end-labeled primer (Pr_24nt^FAM^) (Table 2). The FAM–Pr24 DNA primer extension reaction was performed at 72 °C for 30 s by mixing equimolar amounts of DNA polymerase and annealed FAM–Pr24/M44 substrate, followed by the addition of a stop solution (95% formamide, 10 mM EDTA, 10 mM KOH, 2 μM R44, and 0.05% xylene cyanol). The samples were denatured by heating at 100 °C for 5 min followed by cooling on ice. R44, which has the same sequence as the fully extended primer but lacks a fluorophore, prevented significant rehybridization of the fluorescent products with the DNA template. The polymerase reaction products were analyzed by gel electrophoresis in 15% PAAG via a Mini-PROTEAN vertical chamber (Bio-Rad Laboratories, Inc., Hercules, CA, USA) at 200–250 V. The gel was visualized using the VersaDoc gel-documenting system (Bio-Rad Laboratories, Hercules, CA, USA). The conversion degree was obtained by analyzing the electropherogram, calculating the ratio of the sum of the peak areas of the extension products to the sum of the peak areas of the products and the original oligodeoxyribonucleotide. The determined half-life was 9.1 ± 0.3 h.

### 2.7. PCR Efficiency Assay

Fragments 2-kb in length were amplified using λ–Anchor–F and λ–2 kb–R primers with Tst(wt) and TstP36H–Sso7d. PCR amplification was carried out as follows: one initial denaturation step at 94 °C for 30 s; 30 cycles of 94 °C for 30 s, 60 °C for 30 s, 72 °C for 5, 10, 30, 60, and 90 s using λ phage DNA as a template. For long-range PCR, a set of primers (Table 2) was used to amplify amplicons 2–20 kb in size. PCR amplification was carried out as follows: one initial denaturation step at 94 °C for 30 s; 30 cycles of 94 °C for 30 s, 60 °C for 30 s, 72 °C for 1, 1.5, 2, 5, 10 min, and 1 cycle of 72 °C for 10 min using λ phage DNA as a template. In all cases, a 20 µL PCR mixture contained 20 ng of λ phage DNA, 0.2 µM of each primer, 2 U of each DNA polymerase, and 1× the reaction buffer1. For PfuV93Q–Sso7d DNA polymerase, the PfuSE buffer was used, containing 20 mM of Tris-HCl (pH 8.8 at 25 °C), 10 mM of KCl, 10 mM of (NH_4_)_2_SO_4_, 2 mM of MgSO_4_, and 0.1% Triton X-100 (SibEnzyme, Novosibirsk, Russia).

### 2.8. Steady-State Kinetic Assays

The steady-state kinetic experiments were performed to examine the kinetics associated with incorporating a correct nucleotide onto a primer–template duplex. The DNA 44-mer template (Template_44nt) was annealed to the 24-mer FAM 5′ end-labeled primer (Pr_24nt^FAM^). Moreover, 50 nM of the template–primer complex was extended with TstP36H–Sso7d and PfuV93Q–Sso7d DNA polymerases (1 µM) at 20 °C in reaction buffer1 and PfuSE buffer (SibEnzyme), respectively. The dNTP concentration varied from 2 to 20 µM for TstP36H–Sso7d DNA polymerase and from 10 to 500 µM for PfuV93Q–Sso7d DNA polymerase. Moreover, 10 µL aliquots were taken from the reaction mixture at different time points. The enzymatic reaction was quenched with an equal volume of stop buffer (95% formamide, 10 mM EDTA, 10 mM NaOH, 2 μM of a competitor oligodeoxynucleotide template_44nt_R, and a 0.05% xylene cyanol indicator dye). The primer–templates were denatured by heating at 100 °C for 5 min followed by cooling on ice. The competitor, which has the same sequence as the fully extended primer but lacks the fluorophore, prevents significant rehybridization of the fluorescent products to the template. Product separation via electrophoresis was carried out in 15% PAAG under denaturing conditions (7 M urea) in Mini-PROTEAN chambers (Bio-Rad Laboratories, Inc., Hercules, CA, USA) with a voltage of 200–300 V. The gel was visualized by means of a VersaDoc gel-documenting system (Bio-Rad Laboratories, Hercules, CA, USA). The experiments to assess the incorporation of incorrect nucleotides were performed at 20, 37, and 56 °C; the dNTP concentration was 10 mM for TstP36H–Sso7d DNA polymerase and 5 mM for PfuV93Q–Sso7d DNA polymerase. For the PfuV93Q–Sso7d DNA polymerase, the PfuSE buffer was used, comprising 20 mM of Tris-HCl (pH 8.8 at 25 °C), 10 mM of KCl, 10 mM of (NH_4_)_2_SO_4_, 2 mM MgSO_4_, and 0.1% Triton X-100 (SibEnzyme, Novosibirsk, Russia).

The product formation at each time point was computed as the ratio of the sum of the peak areas of extension products to the sum of the peak areas of the products and of a starting oligodeoxyribonucleotide. The kinetic data were analyzed using nonlinear regression. Equations were generated using OriginLab 8.0 software (OriginLab Corp., Northampton, MA, USA). Data points obtained during the experiment were fitted to a single–exponential equation to calculate the observed rate constant (*k*_obs_). The dependence of *k*_obs_ values on the dNTP concentration was used to determine *K*^dNTP^_m_, the dissociation constant for binding dNTP to the [enzyme·primer/template] binary complex, and *k*_cat_, the maximum rate of chemical catalysis. This was achieved by fitting the dependence of the observed rate constants on the dNTP concentration to the following quadratic equation (Equation (1)) [32,33,34]:

(1) $$k_{obs}=\frac{k_{cat}\times [dNTP]}{K_{m}^{dNTP}+[dNTP]}$$

## 3. Results and Discussion

### 3.1. DNA Polymerase Gene

The original Tst(wt) DNA polymerase gene encompassed 2328 bp and encoded the ‘775 amino acids’ protein with an estimated molecular mass of 89.9 kDa. Alignment of several related amino acid sequences was performed using EMBL–EBI sequence analysis tools [35], available in the NCBI database, namely KOD1, Tgo, Tfu, and Tli DNA polymerases from *Thermococcus,* and Pfu DNA polymerase from *Pyrococcus* (Table 3). Amino acid sequence alignment revealed that the Tst(wt) DNA polymerase contained highly conserved motifs of the archaeal family B DNA polymerases, including the three 3′ → 5′ exonuclease motifs [36], six 5′ → 3′ polymerase motifs [37], and the DNA-binding motif Y–G(G/A) [38]. The presence of the highly conserved motif in Tst(wt) DNA polymerase suggested 3′ → 5′ exonuclease and 5′ → 3′ polymerase activity. The multiple sequence alignment showed that Tst shared 93.93 and 92.38% sequence similarity with KOD1 and Tgo, respectively, and 88.40% with Tfu. The similarity with Tli and Pfu reached 77.43 and 78.91%, respectively.

### 3.2. TstP36H–Sso7d DNA Polymerase

The creation of a fusion enzyme with the thermostable DNA-binding protein Sso7d from *Sulfolobus solfataricus* or Sac7d from *Sulfolobus acidocaldarius* is one of the most common modifications used to increase the processivity of family B DNA polymerases [39,40]. These proteins have high thermal and chemical stability and effectively bind DNA without preference in the binding site. The fusion of Sso7d and Sac7d with DNA polymerases from families A and B increases their processivity without compromising the catalytic activity and stability of the enzymes [40].

One of the characteristics of family B DNA polymerases from archaea is their ability to recognize unrepaired uracil in the DNA template, which leads to blocking the replication process. The ability to recognize and bind deaminated bases is due to the presence of a specialized “U-binding pocket” (amino acid residues 7; 36–37; 90–93; 111–116; 119; 123), located in the N-terminal domain [41,42]. The amino acid residues that form this pocket are highly conserved and provide specific interactions with the deaminated base. DNA replication is blocked, and the repair systems correct the error. Under U-binding, the DNA backbone is distorted and the uracil base is located deep inside the pocket. The ability of archaeal DNA polymerases to recognize unreduced uracil is apparently protective against increased levels of cytosine deamination; however, for PCR, this property can lead to a decrease in the yield of DNA amplification and a decrease in sensitivity. Using rational design, mutant forms of family B DNA polymerases were obtained that are capable of “passing” uracil in the template [41,42,43]. The most successful amino acid substitutions are considered to be Pro36His, Tyr37Phe, and Val93Gln [41,43]. Mutant form V93Q of Pfu DNA polymerase is most often used in biotechnological applications. The Val93 residue is located in a hydrophobic α-helix (amino acid residues 90–97) that forms one side of the U-binding pocket. The V93Q substitution reduces the enzyme’s affinity for uracil-containing DNA and dUTP in the reaction medium by more than tenfold [41,43]. For DNA polymerase Sh1B from *Thermococcus litoralis,* it was shown that P36H amino acid substitution [43] completely switched off the binding of uracil while maintaining the polymerase’s fidelity and structural integrity; thus, we used this amino acid substitution in the Tst DNA polymerase to impart “U-resistant” properties.

By site-directed mutagenesis, a point mutation P36H was introduced into the Tst(wt) DNA polymerase gene to reduce the enzyme’s affinity for the U-containing template and dUTP in the reaction medium. Also, the nucleotide sequence coding the DNA-binding protein Sso7d from *S. solfataricus* was inserted into the DNA polymerase gene to produce the final chimeric form of TstP36H–Sso7d.

### 3.3. Purification of Tst DNA Polymerases

**Figure 1:**
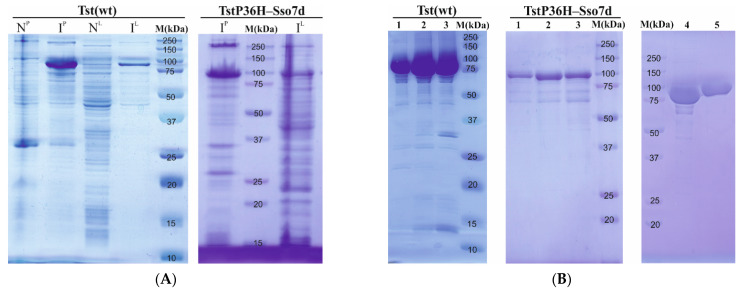
Expression and purification assay of Tst DNA polymerases. (**A**) Expression analysis of Tst(wt) and the TstP36H–Sso7d DNA polymerase gene in *E. coli* Rosetta 2(DE3) pLysS cells containing the recombinant plasmids. A. Lane M, protein molecular mass markers; Lane N, sonicated pellet (N^P^) and lysate (N^L^) of uninduced cells; Lane I, sonicated pellet (I^P^) and lysate (I^L^) of induced cells. (**B**) Purification of Tst(wt) and TstP36H–Sso7d DNA polymerases. Lines 1–3 indicate protein-containing fractions from chromatographies in a HiTrap Heparin™ column, Line 4, resulting in the Heparin-purified Tst(wt) DNA polymerase, Line 5, resulting in the phenyl-purified TstP36H–Sso7d DNA polymerase.

**Figure 2:**
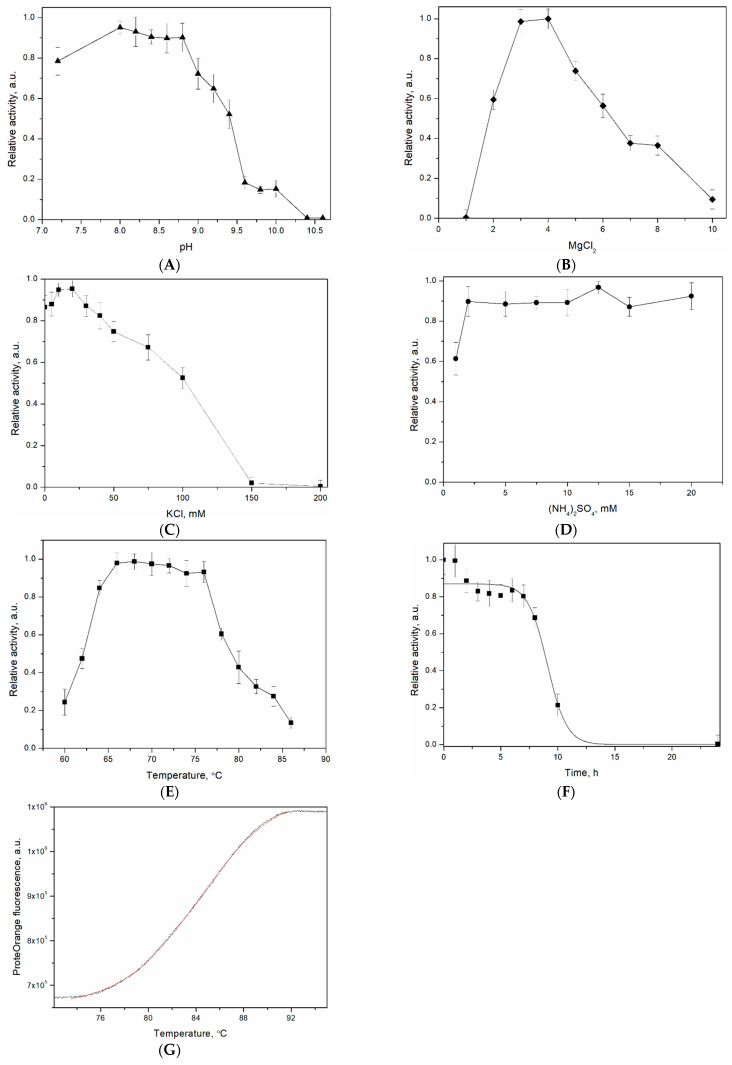
Characterization of the Tst(wt) DNA polymerase. DNA polymerase activity of the purified Tst(wt) DNA polymerase assayed under the indicated conditions. The TstP36H–Sso7d DNA polymerase showed the same results; therefore, these results are presented as one. Effects on Tst(wt) DNA polymerase activity in terms of (**A**) pH; (**B**) MgCl_2_; (**C**) KCl; (**D**) (NH_4_)_2_SO_4_; and (**E**) elongation temperature. (**F**) Thermostability of Tst(wt) DNA polymerase; the purified enzyme was incubated at 95 °C. (**G**) The DSF profile, black trace represents experimental data, red curve is theoretically fitted.

The recombinant Tst(wt) and TstP36H–Sso7d DNA polymerase genes were expressed under the control of the T7lac promoter of pET–28c in *E. coli* Rosetta 2(DE3) pLysS cells. The production levels of the Tst(wt) and TstP36H–Sso7d DNA polymerases were detected by SDS–PAGE (Figure 1A). Tst(wt) and TstP36H–Sso7d DNA polymerases were expressed with a 6xHis-tag and thrombin cleavage site in the N-terminus. Thus, both proteins were subjected to an initial two-step purification procedure: immobilized metal ion affinity chromatography (IMAC) using a Ni Sepharose^TM^ High-Performance resin followed by cation-exchange column chromatography at a HiTrap Heparin™ column. To further ensure a pure protein product, TstP36H–Sso7d DNA polymerase was eluted from a HiTrap Phenyl HP^TM^ column at low salt concentrations. The purity of DNA polymerases was monitored by SDS–PAGE electrophoresis (Figure 1B). The Tst(wt) DNA polymerase revealed a major protein band of approximately 90 kDa, consistent with a molecular mass of 92,399.19 Da, calculated based on the 796 amino acid sequence. And the TstP36H–Sso7 DNA polymerase revealed a major protein band of approximately 100 kDa, consistent with a molecular mass of 99,915.9 Da, calculated based on the 865 amino acid sequence. Approximately 20.9 mg of the purified Tst(wt) DNA polymerase and 4.3 mg of the purified TstP36H–Sso7d DNA polymerase after Heparin purification were recovered from a 1 L bacterial culture. The additional purification of the TstP36H–Sso7d DNA polymerase with the HiTrap Phenyl HP^TM^ column resulted in an ultra-pure protein fraction (Figure 2B) with a significant loss of protein yield—0.6 mg from a 1 L bacterial culture. The optimization of induction conditions enabled the use of a one-step (metal ion affinity chromatography) or two-step (metal ion affinity chromatography followed by cation–exchange column chromatography) purification protocol for both enzymes. As the main goal of the present study involved the characterization of Tst DNA polymerase biochemical properties and their possible use in PCR, the high purity level of obtained enzymes justifies the resources expended.

### 3.4. Characterization of Tst DNA Polymerases

The reaction buffer optimization can improve the PCR yield and specificity. Therefore, the optimal PCR buffer for the Tst(wt) DNA polymerase was determined. A 2 kb λ DNA fragment was optimally amplified at pH 8.0–8.6 (Figure 2A). Moreover, PCR was carried out with various concentrations of MgCl_2_, KCl, and (NH_4_)_2_SO_4_, resulting in optimal concentrations of 3–4 mM MgCl_2_ (Figure 2B), 20 mM KCl (Figure 2C), and up to 20 mM (NH_4_)_2_SO_4_ (Figure 2D). Each buffer component was successively modified during the characterization of the DNA polymerase activity. Triplicate experiments established that the optimal PCR buffer for the Tst(wt) DNA polymerase was 20 mM of Tris–HCl (pH 8.4), 20 mM of KCl, 3 mM of MgCl_2_, and 12.5 mM of (NH_4_)_2_SO_4_). 

The maximal activity of Tst(wt) DNA polymerase in PCR conditions was observed at a temperature range from 66 to 76 °C (Figure 2E). The thermostability of Tst(wt) DNA polymerase was examined by measuring the decrease in activity after preincubation at 95 °C (Figure 2F). The half-life of the enzyme at 95 °C was about 9.1 h.

The melting temperature of the Tst(wt) DNA polymerase was studied using differential scanning fluorimetry (DSF). DSF is based on the ability of some fluorescent dyes to bind with hydrophobic protein regions. Typically, during thermal denaturation, soluble globular proteins undergo structural rearrangement, which leads to the greater accessibility of the internal hydrophobic regions of the protein globule for dye-binding. The binding of hydrophobic residues to a fluorescent dye leads to an increase in the intensity of the fluorescent signal. A change in protein stability is indicated by a shift in the thermal profile. The DSF was successfully used for the thermal stability determination of family B, Bst, and other DNA polymerases [32,44]. As family B DNA polymerases are extremely thermostable with the unfolding transition being incomplete at 100 °C, the addition of guanidinium hydrochloride makes thermal unfolding more accessible [32]. The melting profile of the Tst(wt) DNA polymerase is shown in Figure 2G; the melting transition was observed at a Tm of 84.3 ± 0.9 °C, demonstrating good agreement with Pfu and KOD1 DNA polymerases [32].

### 3.5. Comparison Between PCR Efficiency of the Tst and PfuV93Q–Sso7d DNA Polymerases

**Figure 3:**
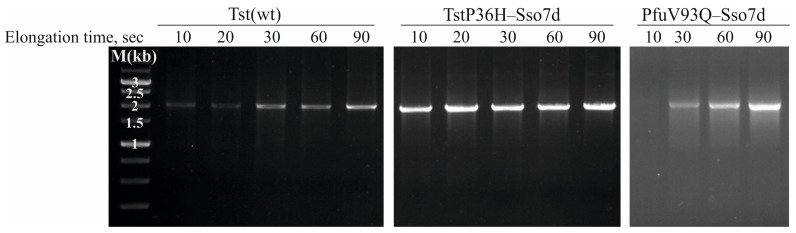
Comparison of PCR efficiency in amplifying a 2-kb fragment from λ DNA using Tst(wt), TstP36H–Sso7d, and PfuV93Q–Sso7d DNA polymerases with various extension times. The enzymes used and extension times are indicated at the top of the figure. PCR products were resolved using 1.0% agarose gel electrophoresis. Lane M, Sky-High DNA ladder (Biolabmix LLC, Novosibirsk, Russia).

The PCR efficiencies of the Tst(wt) and TstP36H–Sso7d DNA polymerases in terms of faster and/or longer amplifications were determined. To investigate the efficiencies of Tst(wt) and TstP36H–Sso7d DNA polymerases for minimum extension times (10 s, 20 s, 30 s, 60 s, and 90 s), PCR was carried out for a 2-kb DNA fragment using the optimal PCR buffer1 determined above. As can be seen, the Tst(wt) DNA polymerase amplified DNA more rapidly relative to the PfuV93Q–Sso7d DNA polymerase, as the Tst(wt) DNA polymerase can amplify a pronounced 2-kb DNA fragment in 10 s, whereas PfuV93Q–Sso7d DNA polymerase did not amplify the same DNA target at the same time. It should be noted that the TstP36H–Sso7d DNA polymerase demonstrated a higher PCR efficiency than those of Tst(wt) and PfuV93Q–Sso7d DNA polymerases (Figure 3), and a 10 s extension time is enough for the 2-kb fragment amplification.

**Figure 4:**
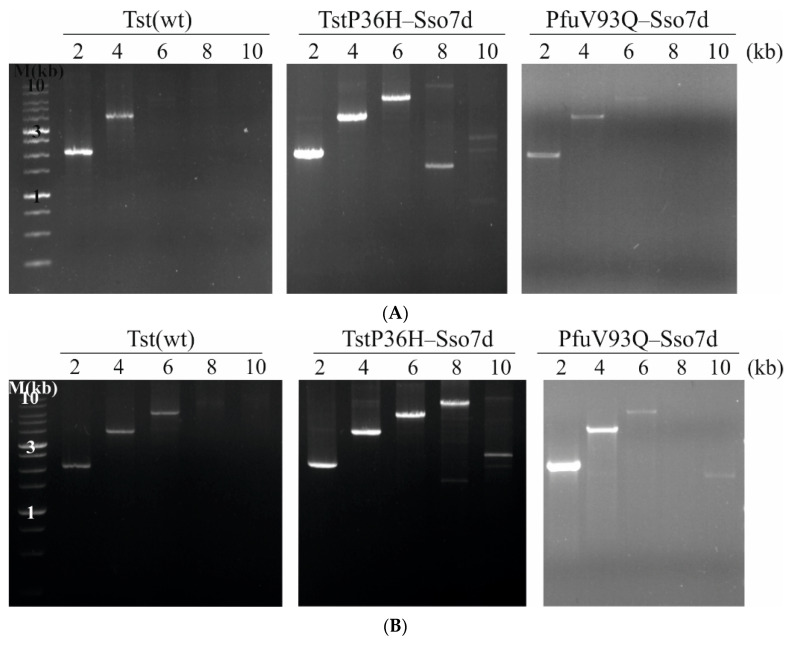

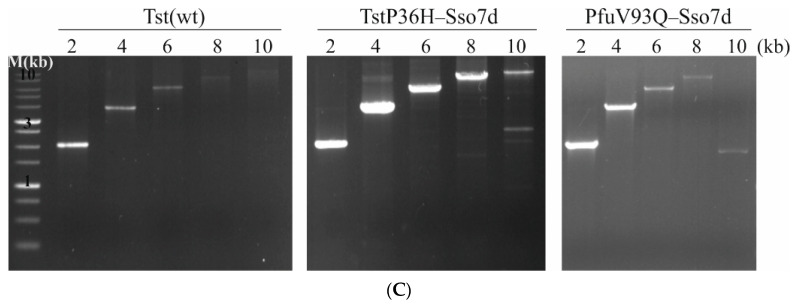
Comparison of PCR amplification of 2–10 kb from λ DNA using Tst(wt), TstP36H–Sso7d, and PfuV93Q–Sso7d DNA polymerases with various extension times. Amplicon sizes are indicated at the top of the figure. Lane M contains DNA molecular-sized markers (SkyHigh DNA ladder, Biolabmix, LLC, Novosibirsk, Russia). The cycling protocol consisted of one initial denaturation step at 94 °C for 30 s; 30 cycles of 94 °C for 30 s, 60 °C for 30 s, 72 °C for 1 min (**A**), 1.5 min (**B**), 2 min (**C**), and 1 cycle of 72 °C for 10 min.

To investigate the efficiencies of Tst(wt) and TstP36H–Sso7d DNA polymerases in the amplification of different-sized DNA fragments, PCR was performed using 2-, 4-, 6-, 8-, and 10-kb DNA fragments with different primer combinations (Figure 4). To compare the relative enzyme’s efficacy, the extension times were 60 s (Figure 4A), 90 s (Figure 4B), and 120 s (Figure 4C). As can be seen, the Tst(wt) DNA polymerase can affective amplify 2–6-kb DNA fragments, whereas TstP36H–Sso7d DNA polymerase can effectively amplify up to 10-kb DNA fragments within 2 min. It should be noted that the PCR efficiency of TstP36H–Sso7d DNA polymerase was generally better than that of the PfuV93Q–Sso7d DNA polymerase.

**Figure 5:**
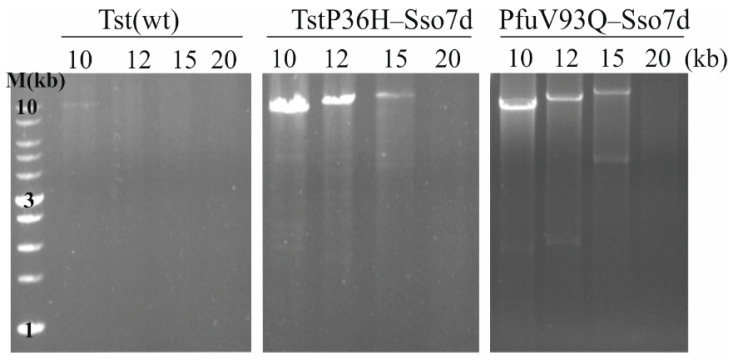
Comparison of PCR amplification of 10–20-kb from λ DNA using Tst(wt), TstP36H–Sso7d, and PfuV93Q–Sso7d DNA polymerases. Amplicon sizes are indicated at the top of the figure. Lane M contains DNA molecular-sized markers (SkyHigh DNA ladder, Biolabmix, LLC, Novosibirsk, Russia). The cycling protocol consists of one initial denaturation step at 94 °C for 30 s; 30 cycles of 94 °C for 30 s, 60 °C for 30 s, 72 °C 5 min, and 1 cycle of 72 °C for 10 min.

The limitation of most used thermostable DNA polymerases is the difficulty in amplifying long target DNAs due to the low yield of amplification products over 10 kb. For PCR amplification of DNA fragments up to 35 kb, a combination of 3′ → 5′ exonuclease-free thermophilic pol I DNA polymerase (Kletaq1) and family B DNA polymerases (Pfu and Tli) can be used [45]. In order to determine the size limit of DNA fragments amplified by Tst(wt) and TstP36H–Sso7d DNA polymerases, the amplification of 10–20-kb DNA fragments with different primer combinations was performed (Figure 5). As can be seen, TstP36H–Sso7d and PfuV93Q–Sso7d DNA polymerases can amplify DNA fragments of up to 15 kb with an extension time of 5 min. The increased extension time did not result in a dramatic change in the observed picture: the increase in the amount of the PCR product—but not the length—was observed. Nevertheless, the PCR efficiency of TstP36H–Sso7d DNA polymerase was generally better than that of PfuV93Q–Sso7d DNA polymerase in the amplification of long DNA fragments. As expected, Tst(wt) DNA polymerase amplified 10-kb DNA fragments with low efficacy.

### 3.6. Kinetic Analysis of TstP36H–Sso7d DNA Polymerases

**Figure 6:**
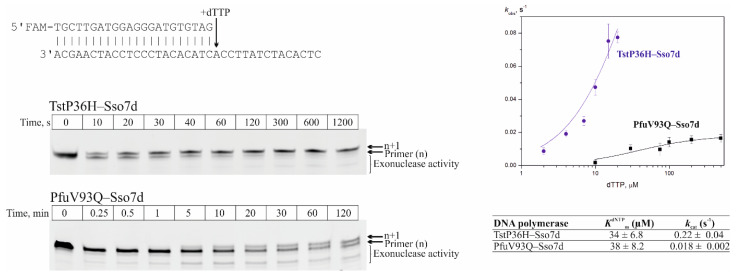
Primer–template elongation using TstP36H–Sso7d and PfuV93Q–Sso7d DNA polymerases under 20 °C. Representative single-nucleotide (dTTP) primer strand extension PAAG analysis with 10 µM dTTP. The nonlinear regression fit of the single-nucleotide extension experiments by the Michaelis–Menten equation for TstP36H–Sso7d (blue) and PfuV93Q–Sso7d (black) DNA polymerases.

We employed a steady-state kinetic assay at 20 °C to compare TstP36H–Sso7d and PfuV93Q–Sso7d DNA polymerases in terms of the dNTP binding affinity (*K*^dNTP^_m_) and the maximum rate of dNTP incorporation (*k*_cat_). For the steady-state kinetic experiments, the DNA 44-mer template annealed to the 24-mer FAM 5′ end-labeled primer. Quantifying the products of a similar single-nucleotide extension reaction and fitting them to the Michaelis–Menten equation allowed us to obtain the dNTP binding affinity (*K*^dNTP^_m_) and the maximum rate of dNTP incorporation (*k*_cat_) (Figure 6). As can be seen in Figure 6, TstP36H–Sso7d DNA polymerase extended primers significantly more rapidly than the PfuV93Q–Sso7d DNA polymerase. The value of *K*^dNTP^_m_ was the same for both DNA polymerases (with an average mean of 36.2 µM), whereas the value of *k*_cat_ was deferred by two orders of magnitude.

**Figure 7:**
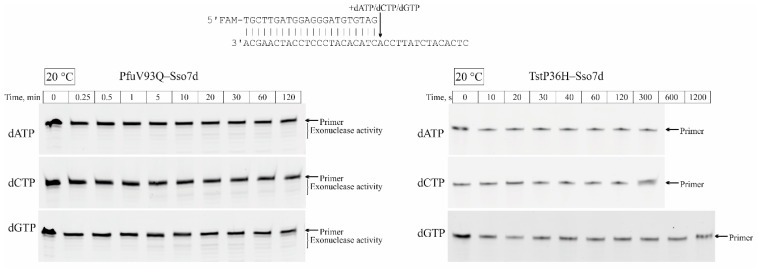

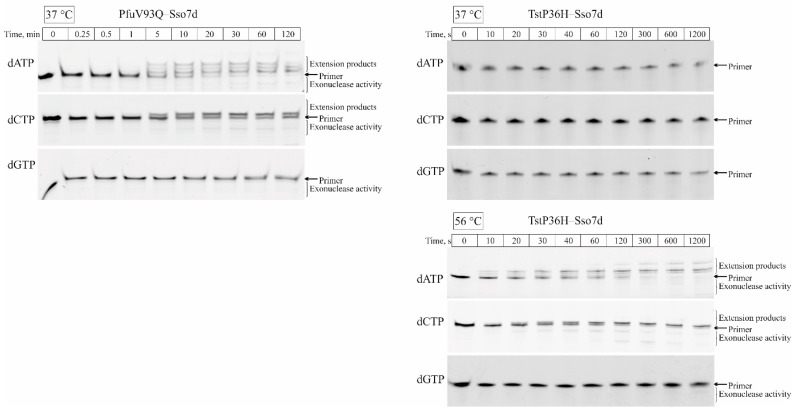
Primer–template elongation by TstP36H–Sso7d and PfuV93Q–Sso7d DNA polymerases at different temperatures with incorrect dNTP. Representative nucleotide (dNTP) primer strand extension PAAG analysis was conducted with 5 mM of dNTP (PfuV93Q–Sso7d DNA polymerase) and 10 mM of dNTP (TstP36H–Sso7d DNA polymerase).

The incorporation of incorrect dNTP was not observed at 20 °C for both DNA polymerases due to the low rates under such conditions according to the van ’t Hoff equation [46]. Under the increase of a reaction temperature up to 37 °C, the formation of extension primer products with incorrect dNTP was observed for the PfuV93Q–Sso7d polymerase but not for TstP36H–Sso7d DNA polymerase (Figure 7). Increasing the reaction temperature to 56 °C allowed us to register extension primer products with incorrect dNTPs for the TstP36H–Sso7d DNA polymerase. This observation suggests that the TstP36H–Sso7d DNA polymerase has a higher selectivity for the incorporation of correct/incorrect dNTPs compared to the PfuV93Q–Sso7d DNA polymerase. 

## 4. Conclusions

This study is the first to report data concerning the purification and biochemical characteristics of the Tst DNA polymerase from *Thermococcus stetteri*. The characterization of the wild type Tst(wt) DNA polymerase and its chimeric form (representing the TstP36H mutant form joined to the DNA-binding protein Sso7d from *S. solfataricus*) was performed. The P36H point mutation was introduced to reduce the enzyme’s affinity for the U-containing template and dUTP in the reaction medium. The well-characterized PfuV93Q–Sso7d DNA polymerase was used as a reference. 

Tst DNA polymerase can be assumed to be an easy enzyme to use in PCR amplification. It is not very sensitive to buffer composition. Amplification of DNA fragments was observed at a wide diapason of pH values (8.0–8.6) and (NH_4_)_2_SO_4_ concentrations (up to 20 mM). The optimal buffer contains 20 mM Tris–HCl (pH 8.4), 20 mM KCl, 3 mM MgCl_2_, and 12.5 mM (NH_4_)_2_SO_4_. The extremely high thermal stability of the studied enzymes is also convenient to use in the PCR. It was shown that Tst(wt) DNA polymerase could effectively amplify up to 6-kb λ DNA fragments, whereas for the TstP36H–Sso7d DNA polymerase, the size limit of amplified DNA fragments was 15 kb. It was confirmed that the TstP36H–Sso7d DNA polymerase has superior PCR efficiency when compared with the PfuV93Q–Sso7d DNA polymerase. Under amplification of the 2-kb DNA fragment, the TstP36H–Sso7d DNA polymerase required less than 10 s for extension, whereas for the PfuV93Q–Sso7d DNA polymerase, the required extension time was no less than 30 s. The steady-state kinetic assay has shown that the dNTP binding affinity (*K*^dNTP^_m_) was the same for TstP36H–Sso7d and PfuV93Q–Sso7d DNA polymerases, whereas the maximum rate of dNTP incorporation (*k*_cat_) was two orders of magnitude higher for the TstP36H–Sso7d DNA polymerase. Moreover, the incorporation of incorrect dNTP was not observed up to 56 °C for the TstP36H–Sso7d DNA polymerase, whereas for the PfuV93Q–Sso7d DNA polymerase, primer extension with incorrect dNTPs was observed at 37 °C. Taken together, these results suggest that the Tst DNA polymerase may be a good candidate for high-fidelity DNA amplification.

## Figures and Tables

**Table 1:** Primer sequences for the cloning and site-directed mutagenesis of the Tst DNA polymerase.

Primer Name	Primer Sequence (5′→3′)
Tst_F	GGACCATATGATCCTCGACACAGACTACATAACCG
Tst_R	GTGCGGCCGCTCACCCCTTCCCCTTCGGC
Tst–P36H_F	5′–GACTACGATAGGACTTTCGAACATTACTTCTACGCCCTTCTGAAGG–3′
Tst–P36H_R	5′–CCTTCAGAAGGGCGTAGAAGTAATGTTCGAAAGTCCTATCGTAGTC–3′
Tst–SSo7d_F	CTGGCTGAAGCCGAAGGGGAAGGGGGGTACCGGCGGTGGCGGTGCA ACCGTAAAGTTCAAGTACAAAGGCGAAGAAAAAGAGGTAGACATCT CCAAGATCAAGAAAGTATGGCGTGTGGGCAAGATGATCTCCTTCACC TACGACGAGGGCGGTGGCAAGACCGGCCGTGGTGCGGTAAGCGAAA AGGACGCGCCGAAGGAGCTGCTGCAGATGCTGGAGAAGCAGAAAAA GTGAGCGGCCGCGGTACCGG
Tst–SSo7d_R	CCGGTACCGCGGCCGCTCACTTTTTCTGCTTCTCCAGCATCTGCAGCA GCTCCTTCGGCGCGTCCTTTTCGCTTACCGCACCACGGCCGGTCTTGC CACCGCCCTCGTCGTAGGTGAAGGAGATCATCTTGCCCACACGCCAT ACTTTCTTGATCTTGGAGATGTCTACCTCTTTTTCTTCGCCTTTGTACT TGAACTTTACGGTTGCACCGCCACCGCCGGTACCCCCCTTCCCCTTCG GCTTCAGCCAG

**Table 2:** DNA substrate oligonucleotide sequences for gel activity assays and PCR primers used for PCR extension efficiency assays with λ DNA.

Primer Name (Target Size)	Primer Sequence (5′→3′)	λ DNA Sequence (bp)
λ–Anchor–F	CCTGCTCTGCCGCTTCACGCAGTGC	30,352–30,376
λ–2 kb–R (2 kb)	CCATGATTCAGTGTGCCCGTCTGG	32,326–32,349
λ–4 kb–R (4 kb)	CCAGGACTATCCGTATGACTACG	34,286–34,315
λ–6 kb–R (6 kb)	GAGATGGCATATTGCTACGCAAGA	36,339–36,362
λ–8 kb–R (8 kb)	GCCTCGTTGCGTTTGTTTGCACG	38,373–38,395
λ–10 kb–R (10 kb)	GCACAGAAGCTATTATGCGTCCCCAGG	40,316–40,342
λ–12 kb–R (12 kb)	TCTTCCTCGTGCATCGAGCTATTCGG	42,401–42,426
λ–15 kb–R (15 kb)	CTTGTTCCTTTGCCGCGAGAATGG	45,220–45,243
λ–Anchor–20F	GATACGGGAAAACGTAAAACCTTCG	1755–1779
λ–20 kb–R	GACTGTCCGTTTTCGATAAATAAGC	21,728–21,752
Pr_33nt	CTCTGTACGTTGGTCCTGAAGGAGGATAGGTTG	
Template_90nt	CCGTCAGCTGTGCCGTCGCGCAGCACGCGCCGCCGTGGACAGAG GACTGCAGAAAATCAACCTATCCTCCTTCAGGACCAACGTACAGAG	
Template_44nt	GGAGACATTTTGCCTTGATAGCTGCTCGACTCATCTGGGGGCCG	
Template_44nt_R	CGGCCCCCAGATGAGTCGAGCAGCTATCAAGGCAAAATGTCTCC	
Pr_24nt^FAM^	FAM–CGGCCCCCAGATGAGTCGAGCAGC	

**Table 3:** Comparison of amino acid sequences of family B DNA polymerases. Identity values were calculated using EMBL–EBI sequence analysis tools.

Organism	Amino Acid Identity (%)					
	Tst	KOD1	Tgo	Tfu	Tli	Pfu
*Thermococcus stetteri*	100	93.93	92.38	88.40	77.43	78.91
*Thermococcus kodakaraensis*		100	90.58	69.03	72.27	79.72
*Thermococcus gorgonarius*			100	94.05	75.83	80.36
*Thermococcus fumicolans*				100	58.20	76.70
*Thermococcus litoralis*					100	74.35
*Pyrococcus furiosus*						100

## Data Availability

Data are available upon request A.A.K. Tel. +7-(383)363-5174, E-mail: sandra-k@niboch.nsc.ru.
